# Does the Patient’s Sex Have an Impact on Beneficial Effects of *Ginkgo biloba* Extract EGb 761^®^ in Dementia Patients with Tinnitus? Results of a Conditional Process Analysis

**DOI:** 10.3390/jcm14176313

**Published:** 2025-09-06

**Authors:** Petra Brueggemann, Marília Grando Sória, Sandra Schlaefke, Petra Funk, Birgit Mazurek

**Affiliations:** 1 Tinnitus Center, Charité–Universitätsmedizin Berlin, 10117 Berlin, Germany; petra.brueggemann@charite.de; 2 Research & Development, Dr. Willmar Schwabe GmbH & Co. KG, 76227 Karlsruhe, Germany; marilia.soria@schwabe.de (M.G.S.); sandra.schlaefke@schwabe.de (S.S.); petra.funk@schwabe.de (P.F.)

**Keywords:** tinnitus, dementia, depression, anxiety, *Ginkgo biloba*, EGb 761^®^, conditional process analysis, mediation, moderation, sex

## Abstract

**Background/Objectives**: Tinnitus often occurs alongside the emotional symptoms of anxiety and depression. The *Ginkgo biloba* extract EGb 761^®^ was shown to be effective in reducing neuropsychiatric symptoms in elderly patients with both dementia and tinnitus, exerting direct effects on tinnitus severity and indirect effects mediated by improvement of anxiety, depression, and cognition. Whether the extent of the effects is influenced by the patient’s sex has not yet been investigated. We performed a conditional process analysis to evaluate this question. **Methods**: We analyzed the moderating role of sex on the direct and indirect effects of EGb 761^®^ on tinnitus severity using a first- and second-stage conditional process model. **Results**: Indirect effects of EGb 761^®^ on tinnitus severity mediated by improved cognition, anxiety, and depression did not differ between women and men (*p* > 0.05; all 95% bootstrap confidence intervals overlapped with zero). Moreover, direct treatment effects of EGb 761^®^ on tinnitus severity were statistically significant for both sexes (women, *p* < 0.0001; men, *p* = 0.0279). **Conclusions**: Beneficial effects of *Ginkgo biloba* extract EGb 761^®^ in dementia patients with tinnitus are likely to be unaffected by the patient’s sex. Further research into the influence of patient characteristics on the outcome of tinnitus drug treatment is encouraged.

## 1. Introduction

Tinnitus is an auditory sensation in the absence of a corresponding source and commonly associated with stress, exposure to noise, loss of hearing, and aging [1]. A recently reported systematic review and meta-analysis estimated the global prevalence of any tinnitus amongst adults to be 14.4%, with no significant difference between men and women, but higher in older adults (around 24%) [2], thus underlining the global burden of tinnitus, especially in the elderly population.

Tinnitus can be categorized as either objective or subjective, differentiated based on comprehensive history, physical examination, and audiogram [3,4]. In most cases, tinnitus is subjective, meaning that it is only perceivable by the individual patient [5]. Tinnitus of this category is usually associated with hearing loss. Objective tinnitus, which constitutes approximately 1.5% of tinnitus cases evaluated in tertiary healthcare institutions [6,7], is defined as a type of tinnitus that is perceived by both the patient and an external observer (e.g., tinnitus due to an aneurysm, temporomandibular joint disease, or tensor tympani muscle spasms) [8].

A higher prevalence of tinnitus has been previously observed in individuals with cognitive impairments, including memory problems and mild cognitive impairment [9,10]. This is in line with recent results from a comprehensive review meta-analysis of 17 studies, which indicated a strong association between tinnitus and a higher risk of dementia-compromised learning, auditory attention, anxiety, depression, and poor sleep quality [11]. Suffering from dementia or cognitive decline, patients may be more susceptible to psychiatric syndromes due to an immediate effect of tinnitus on sleep and because of the focus on and worry about the underlying causes of the disease and, in reverse, patients with anxiety and depression or at risk for these conditions could be more likely to experience tinnitus and tinnitus-related distress [12]. However, a correlation analysis showed the impact of tinnitus on the manifestation of depressive symptoms to be stronger than the reverse [13]. In an epidemiologic study, there was also shown a clear association between tinnitus and a reduced quality of life [14].

It is widely recognized that the perception and evaluation of tinnitus and associated distress involves a whole network of functionally connected brain areas in addition to the auditory cortex [15]. Individuals with tinnitus show significant changes in the connectivity within and between the attention, emotion processing, default mode, and salience networks [15]. These changes may reflect how tinnitus is consciously perceived as well as the attention it receives, its importance, and the distress it causes [16].

Recently, it has been established that the effects of pharmacological treatment on male and female patients can be very different depending on the condition and, therefore, there have been increasing calls for conducting sex-based analyses [17,18,19,20]. Sex is an important determinant of health and well-being and usually categorized as female or male, although there is variation in the biological attributes constituting sex and in how these attributes are expressed [17]. In contrast to the term gender, which refers to the socially constructed roles, behaviors, and identities of female, male, and gender-diverse people, the term sex is used to denote a set of biological attributes that are associated with physiological and physical features including chromosomes, gene expression, the function of hormones, and reproductive/sexual anatomy [17]. As concerns medical treatment, inevitable differences can exist between men and women in terms of efficacy, long-term treatment response, and side effects, which leads, e.g., to the responsibility of healthcare professionals to prescribe sex-specific psychopharmacotherapies to minimize adverse drug reactions, to maximize effectiveness of therapy, and to provide personalized care management [21].

Although the prevalence of tinnitus among men and women is similar [2], relevant sex differences with respect to tinnitus perception and comorbidities are currently being discussed. In a retrospective, explorative analysis of data for differences in patients with tinnitus and those diagnosed with tinnitus and insomnia [22], males had higher depression scores and exhibited more clinically relevant depressive symptoms than females. Women, on the other hand, suffered more from psychosomatic symptoms. The authors concluded that there is a need for targeted therapy, focusing on depressive symptoms in men and on psychosomatic symptoms and stress-related deterioration of insomnia and tinnitus in women. Moreover, results from a large study in patients with chronic tinnitus showed women to report a greater degree of annoyance in relation to tinnitus and perceived more stress compared to men, irrespective of age and tinnitus duration [23]. The study also indicated that women exhibited lower scores than men in proactive coping, sense of coherence, and personal resources, despite having lower levels of hearing loss and tinnitus loudness. The differences were small, yet statistically significant.

As concerns chronic idiopathic tinnitus, German guidelines, in accordance with international guidelines, do not currently recommend any drug treatments in chronic stadium (more than 3 months) [24,25,26,27]. The only exception is the treatment of comorbidities commonly associated with tinnitus, such as anxiety and depression. For these, the respective guidelines may recommend drug therapies [25]. Regardless of the patient’s sex, general therapeutic counseling accentuates coping strategies and reduction of stress [27]. In patients with dementia or cognitive decline in addition to tinnitus of vascular or involutive origin, the *Ginkgo biloba* special extract EGb 761^®^ (EGb 761^®^ is a registered trademark of Dr. Willmar Schwabe GmbH & Co. KG, Karlsruhe, Germany) is a treatment option. EGb 761^®^ is widely used for central nervous system disorders like dementia, age-related cognitive impairment, vestibular and non-vestibular vertigo, and peripheral arterial occlusive disease [12,28] and reported to be the best-researched plant preparation worldwide [29].

EGb 761^®^ is a highly purified dry extract of *Ginkgo biloba* leaves (35–67:1), extraction solvent: acetone 60% (*w*/*w*), adjusted to 22.0–27.0% Ginkgo flavonoids, calculated as Ginkgo flavone glycosides and 5.4–6.6% terpene lactones consisting of 2.8–3.4% ginkgolides A, B, C and 2.6–3.2% bilobalide. An in-depth description of these components, along with their respective molecular structures, has been published elsewhere [30]. Potential mechanisms of action mediating the efficacy of EGb 761^®^ in tinnitus include enhancement of cochlear blood flow, protection against ototoxic stimuli and inhibition of apoptosis, obstruction of age-associated degenerative processes within the inner ear, influence on neurotransmitter actions, effects on neuropsychiatric symptoms, and improvement of cerebral perfusion (for an overview see [28]).

Clinically, moderate improvements in tinnitus loudness and tinnitus-related distress and annoyance were found by randomized controlled trials in dementia patients with tinnitus [31,32]. Results from clinical trials also showed EGb 761^®^ to improve anxiety severity in patients with anxiety disorders [33] and dementia [34], and to reduce symptoms of depression in patients with dementia or mild cognitive impairment, also in the presence of tinnitus [34,35]. In an open-label, multicenter clinical trial exploring whether etiology, risk factors, chronicity, characteristics of tinnitus, or concomitant pathologies are effect modifiers in the outcome of treatment with EGb 761^®^ in patients with chronic tinnitus, a significant improvement in tinnitus loudness and annoyance, assessed by means of 11-point box scales, was seen, with stress level and depression being effect modifiers [36]. Furthermore, results of a retrospective study analyzing the therapeutic effect of add-on therapy of EGb 761^®^ to routine treatment of sudden sensorineural hearing loss and its influence on hemorheology suggest that add-on therapy with EGb 761^®^ may enhance the hearing threshold and hemorheological indexes of patients, whilst concomitantly attenuating the inflammatory response and promoting the recovery of hearing function [37].

Moreover, a recent mediation analysis of data from three randomized placebo-controlled clinical trials [38,39,40] showed that the direct treatment effects of EGb 761^®^ on tinnitus severity in patients suffering from mild to moderate dementia accounted for approximately 60% of the total effect, while the indirect effects mediated by improvement of anxiety, depression, as well as cognition were shown to represent approximately 40% of the total effect [12]. To further add to the evidence of EGb 761^®^, we investigated whether the patient’s sex had an impact on these beneficial direct and indirect effects shown. For this purpose, the data underlying the recent mediation analysis [12] was used to carry out a conditional process analysis.

## 2. Materials and Methods

### 2.1. Data Sources

Analogous to the previous mediation analysis, the present conditional process analysis was based on pooled data from the three randomised placebo-controlled clinical trials published in peer-reviewed journals [38,39,40] and assessing tinnitus and neuropsychiatric symptoms in patients with dementia. The trial populations consisted of patients with mild to moderate dementia (scoring between 9 and 22 points on the SKT Short Cognitive Performance Test [41]). Patients were diagnosed with dementia in accordance with diagnostic criteria of the National Institute of Neurological and Communicative Disorders and Stroke and Alzheimer’ s Disease and Related Disorders Association (NINCDS-ADRDA) [42], or the National Institute for Neurological Disorders and Stroke and Association Internationale pour la Recherche et l’ Enseignement en Neuroscience (NINDS/AIREN) [43], or both. In these trials, tinnitus severity was assessed using an 11-point box scale, depression and anxiety were assessed by the Neuropsychiatric Inventory (NPI), and sex was assessed by means of self-report. More details are described elsewhere [12]. To investigate the influence of sex on the direct or indirect effects of EGb 761^®^ on tinnitus severity, we used the subset of patients suffering from tinnitus, i.e., patients with a tinnitus severity score > 0 points rated on the 11-point box scale (0–10) (n = 594; female, 423; male, 171).

### 2.2. Statistical Analysis

Demographic data (sex, age), baseline scores of tinnitus severity, cognition, anxiety, and depression as well as changes of these scores during treatment were compared between the analyzed treatment groups, using the chi-square (*χ^2^*) test for sex and *t*-tests for all other variables.

Conditional process analysis was used to investigate if sex had an influence on the previously reported direct and indirect effects of EGb 761^®^ on the severity of tinnitus. Conditional process analysis combines mediation analysis and moderation analysis. A mediator is caused by the independent variable and influences the dependent variable. The effect of a moderating variable (moderator) is characterized statistically as an interaction. The moderator is not influenced by the independent variable.

**Figure 1:**
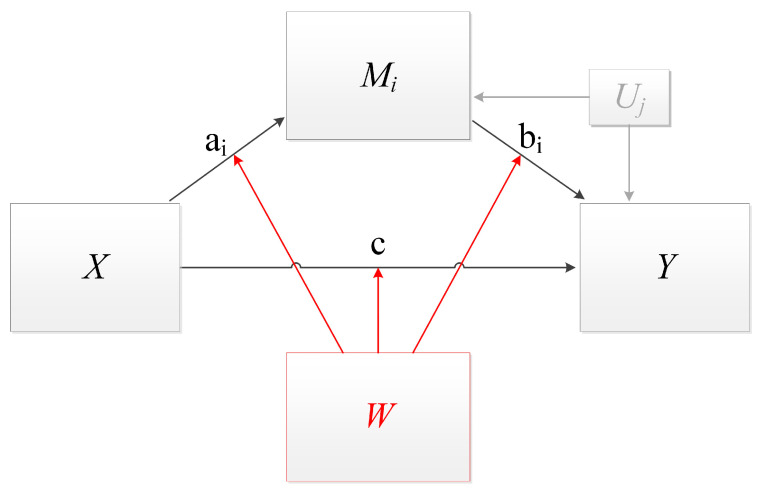
Conditional process model: The process by which the treatment (*X*) affects tinnitus severity (*Y*) through changes of cognition, depression, and anxiety (*M_i_*) is conditional on sex (*W*). Sex influences both the effects of treatment on the changes of cognition, depression, and anxiety and the effects of changed cognition, depression, and anxiety on tinnitus severity. The direct effect of treatment (*X*) on tinnitus severity is influenced by sex. Baseline scores of tinnitus severity, cognition, depression, and anxiety (*U_j_*) affect changes from baseline in tinnitus severity, cognition, depression, and anxiety (*X* = Treatment [EGb 761^®^/placebo]; *M_i_* = mediators [changes from baseline in cognition/depression/anxiety]; *Y*= changes from baseline in tinnitus severity; *U_j_* = baseline values of cognition, tinnitus, depression, anxiety; *W*= moderator [sex]; a_i_ = effect of treatment on mediators, b_i_ = effect of mediators on tinnitus severity, c = direct effect of treatment on tinnitus severity; i = 1, 2, 3; j = 1, 2, 3, 4). The moderating effects of sex (*W*) on direct and indirect effects are printed in red.

The indirect effects of EGb 761^®^ were mediated by improved cognition or symptoms of anxiety or depression (mediators) [12]. The mediation model already described by Brüggemann and colleagues [12] was complemented by the moderator “sex”, and the baseline values of tinnitus severity, cognitive impairment, anxiety, and depression were included as covariates. Baseline values were included to adjust for different baseline characteristics. A first- and second-stage conditional process model [44] (Figure 1) was specified to analyze the moderating role of sex on the direct effect (path c: treatment—tinnitus cfb, cfb = changes from baseline), on the mediators (paths a1, a2, a3: treatment—cognition/depression/anxiety cfb), and on the effect of the mediators on the change of tinnitus severity (paths b1, b2, b3 cognition/depression/anxiety cfb—tinnitus cfb).

The conditional indirect effects of EGb 761^®^ on tinnitus were estimated with standard errors and 95% percentile bootstrap confidence intervals based on 5000 bootstrap samples. If a 95% confidence interval did not include zero, this supported moderation of the indirect effect by sex (*p* < 0.05).

Data analysis was obtained using SAS Version 9.4 (SAS Institute Inc., Cary, NC, USA) and HAYES PROCESS macro model 59 [45] including covariates.

## 3. Results

In the three clinical trials analyzed, a total of 1220 patients suffering from dementia with concomitant neuropsychiatric symptoms were enrolled and randomly assigned to treatment with EGb 761^®^ (n = 611) or placebo (n = 609). Of these, 594 patients (EGb 761^®^, 289; placebo, 305) suffered from tinnitus at the time when the randomized treatment started. For 518/594 patients, depressive or anxious symptoms were documented. The subsequent analyses were based on a total of 594 patients suffering from tinnitus with or without anxious or depressive symptoms. More details are published elsewhere [12]. Demographic data and baseline scores are shown in Table 1. About 70% of the patients (423/594) were women, the mean age was 65 years. The treatment groups were statistically comparable with respect to sex, age, as well as baseline scores of tinnitus, depression, and anxiety. All results described in this section refer to patients with dementia and accompanying tinnitus.

Results of the regression models applied are shown in Table 2. The effect of EGb 761^®^ on the mediators (changes from baseline in cognition, depression, and anxiety) does not depend on the sex of the patients. There are no statistically significant interactions of EGb 761^®^ or placebo treatment with sex on the mediators (*p* > 0.2). Including the interaction of treatment and sex into the regression models resulted in little increased model fit (ɅR^2^ < 0.01).

Neither the direct effects nor the indirect effects of EGb 761^®^ on tinnitus severity were moderated by sex (*p* = 0.4742 for the direct effect; *p* > 0.05 for indirect effects, all 95% bootstrap confidence intervals overlapped with zero) (Table 2 and Table 3). Direct treatment effects of EGb 761^®^ on tinnitus severity were statistically significant for both women (*p* < 0.0001) and men (*p* = 0.0279) in patients with dementia (Table 4). The direct treatment effects (i.e., the differences between EGb 761^®^ and placebo) on tinnitus severity differed between women and men by 0.1725 points on the 11-point box scale for tinnitus severity (Table 2 and Table 4).

The conditional indirect treatment effects on tinnitus severity differed between women and men by 0.0127 to 0.0719 points on the 11-point box scale for tinnitus severity (*p* > 0.05, all 95% bootstrap confidence intervals overlapped with zero) (Table 3). The overlap with zero seen for the calculated 95% bootstrap confidence intervals for the indices of moderated mediation, which quantify the relationship between the moderator (sex) and the indirect treatment effect, indicate that sex did not statistically significantly moderate the indirect treatment effects. EGb 761^®^ did not show a sex-related effect on changes from baseline in cognition, depression, or anxiety. The mediators did not show a sex-related effect on tinnitus severity either.

Baseline scores of cognition, anxiety, and depression significantly influenced the respective changes from baseline in cognition (*p* = 0.0067), depression (*p* < 0.0001), and anxiety (*p* < 0.000). Changes in tinnitus severity from baseline were influenced by the baseline scores of tinnitus (*p* < 0.0001), cognition (*p* = 0.0036), and anxiety (*p* = 0.0115).

## 4. Discussion

The present conditional process analysis examined whether sex influenced the direct or indirect effects of EGb 761^®^ on tinnitus severity in patients with dementia. As memory problems, mild cognitive impairment, and tinnitus share underlying mechanisms, such as neuroinflammation, oxidative stress, and neurotransmitter imbalances [11,46], the chosen patient group was well suited for this conditional process analysis.

Our results show that neither the direct nor the indirect treatment effects of EGb 761^®^ on tinnitus severity shown recently [12] were moderated by the patient’s sex. Between the female and male groups, direct effects on tinnitus severity differed by 0.1725 points and indirect effects by up to 0.0719 points on the 11-point box scale. These slight differences are not considered to be clinically relevant. The absence of sex differences in effects in this study may be due to EGb 761^®^ having multiple pharmacological targets. This hypothesis is supported by an animal experiment in which male and female Alzheimer’s disease model mice (5xFAD) exhibited sex-specific differences in gene expression compared to wild-type mice treated with EGb 761^®^ [47]. However, similar effects on cognition were observed in both sexes.

Previous findings from a cross-sectional study suggest the presence of sex-specific biopsychosocial factors in the occurrence of bothersome tinnitus [48]. In a clinical trial carried out in Hungary and involving 630 patients with chronic tinnitus, tinnitus severity was not affected by duration, localization of the symptoms, and age, but by patient-reported gender, indicating higher values in the case of females [49]. This raised the question of whether treatment response also depends on sex. A data analysis of sex-specific differences regarding the effect of a seven-day multimodal non-pharmacological treatment found that female patients reported a higher response to treatment, higher levels of tension and distress, and better psychological coping [50]. In contrast, chronic tinnitus was associated with higher levels of bodily pain in male patients, despite them rating their general health as better. Sex differences were also found to be an important factor in predicting the outcome of acoustic stimulation therapy [51]. In another study, the outcomes of four different physical tinnitus treatments were combined and compared [52]. Treatment outcome was assessed by the participants via the Tinnitus Functional Index questionnaire at three time points: baseline, immediately after treatment, and after a follow-up period of 9 weeks (±3 weeks). The results suggest that males and females responded differently to the therapy. Significant interactions between sex and time point were observed in all groups except for those receiving combined tinnitus retraining therapy (TRT) and eye movement desensitization and reprocessing (EMDR) therapy. In the groups with high-definition transcranial direct current stimulation (HD-tDCS) or orofacial physical therapy (OPT), females improved significantly more than males. With the combined TRT and Cognitive Behavioral Therapy (CBT), however, male patients with tinnitus significantly improved, while females did not. Another study also showed male patients to respond better to combined TRT and CBT [53]. To our knowledge, however, no clinical study has yet investigated whether the outcome of drug treatment in chronic tinnitus is influenced by the patient’s sex.

Various interrelated psychosocial factors, such as personality traits and stress reactivity, comorbidities like depression or anxiety, and demographic factors like age, sex, and education level, may increase vulnerability to tinnitus-related distress [54]. In both sexes, depression, sleep disturbance, tinnitus frequency, and loudness were found to be associated with tinnitus-related distress [50]. In particular, depression, anxiety, and stress were reported to be significant risk factors for tinnitus [55]. The fact that women may be more susceptible to these comorbidities could explain these sex disparities. Differences of anatomical or physiological factors such as brain circuits, molecular mechanisms, and sexual hormone levels may also be involved [19]. In an ideal world, therapies are individualized to every patient by considering their sex. Since this is not possible due to constraints of limited resources in the healthcare system, the discussion of sex-neutral therapeutic approaches is gaining attention.

To our knowledge, this study is the first to ask whether the effect of a drug—EGb 761^®^ in this case—on patients with dementia and tinnitus is sex-dependent. The data derived from a significant number of patients from multiple studies. Pooling the data was possible because all three trials on which our analysis was based [38,39,40] had similar designs with very similar inclusion criteria and outcome measures. The same trials were also the basis of a preceding mediation analysis, which showed a statistically significant and clinically meaningful reduction of tinnitus scores for EGb 761^®^ compared to placebo [12]. In the applied model with three mediators, the authors correlated changes in tinnitus severity, cognition, anxiety, and depressive symptoms. The prevalence of tinnitus in this cohort of dementia patients was high at almost 50%, underlining the close association of both pathologies, and indirectly the even closer association of hearing loss, which is frequently accompanied by chronic tinnitus and dementia [9,56,57,58]. Based on the results of the mediation analysis, the authors judged that EGb 761^®^ may be regarded as a supporting treatment for tinnitus in elderly patients suffering from dementia, with an added benefit in those experiencing symptoms of depression or anxiety [12]. Our present analysis adds to these findings by showing that this applies regardless of the patient’s sex.

A strength of our analysis was the use of a first- and second-stage conditional process model, which allowed the analysis of the moderating role of sex on direct and indirect effects of first and second stage. Furthermore, the baseline values of the moderators and of tinnitus severity were used as covariates to adjust for different baseline characteristics.

As a limitation of our study, the findings are specific for the group of demented patients and can therefore not be generalized. It should be noted that the utilization of data from earlier clinical trials in this analysis precluded the examination of other potential factors that could have influenced the treatment outcomes. In this context, it would be of interest that subsequent research endeavors focus on the distinctions between tinnitus patients with and without dementia within this particular research question.

Furthermore, the result may be influenced by the fact that, apparently, the perception of tinnitus is different between men and woman. Moreover, concerns may be raised about the reliability of tinnitus self-assessment by patients suffering from mild to moderate dementia. It should be noted, however, that any error in this regard would most likely affect both treatment groups equally. The results of self-reporting for assessment of tinnitus severity and changes during treatment may be less accurate for dementia patients than for cognitively healthy individuals. Nonetheless, it is not to be expected that three large and independent double-blind clinical trials such as those used for the present analysis would have revealed a consistent pattern of superiority over placebo if the self-assessments applied had been unreliable and were predominantly driven by chance. We therefore consider the self-assessments to be reliable.

Finally, it should be kept in mind that there has been debate about the best use of the terms ‘sex’ and ‘gender’. Although the correct use of terms has been established now as sex when referring to biological factors, and gender when reporting gender identity or psychosocial or cultural factors, these terms have not been and are not yet adequately considered across the board in scientific literature. In a recent review of 640 original clinical articles published by renowned medical journals in the year 2020, 97.6% reported sex or gender [59]. However, only 28 (4.7% of all 640 articles) made use of the term ‘gender’, and of these, the majority (23 of 28) did not differentiate between ‘gender’ and ‘sex’, employed ‘gender’ in lieu of ‘sex’, or used it interchangeably with ‘sex’. Recent guidelines therefore demand that authors should provide an explanation as to whether the sex of human research participants was assessed by self-report or was assigned subsequent to an internal or external examination of body characteristics or via genetic testing or other means [17,60]. When sex is assessed by self-report—as was the case in the three clinical trials analyzed—it could be incorrect in very few cases, but, in most studies, carrying out detailed genetic testing in order to determine the participants’ genetic make-up is not possible [61]. However, it is not to be expected that a misclassification in very few cases will lead to a relevant bias of the results.

## 5. Conclusions

Our conditional process analysis suggests that the shown beneficial effects of *Ginkgo biloba* extract EGb 761^®^ in dementia patients with tinnitus are not subject to the patient’s sex. Further research addressing the influence of patient characteristics on the outcome of drug treatment in tinnitus is encouraged.

## Figures and Tables

**Table 1:** Demographic data, baseline scores, and changes during treatment of patients with tinnitus at baseline (overall trial data; absolute (%) frequency with *p*-value of the two-sided *χ^2^* test, or mean (standard deviation) with *p*-value of the two-sided *t*-test, respectively; significance levels of 0.05 with *p*-values < 0.05 highlighted in bold).

Treatment	N	Female	Age (Years) ^1^	Baseline Scores				Change Scores			
				T_BS_	D_BS_	A_BS_	C_BS_	T_CS_	D_CS_	A_CS_	C_CS_
EGb 761^®^	289	209 (72.3)	65.2 (8.9)	3.3 (1.7)	2.1 (2.0)	3.1 (2.4)	16.1 (3.8)	−1.4 (1.6)	−0.7 (1.6)	−1.0 (1.8)	−2.5 (2.7)
Placebo	305	214 (70.2)	65.1 (9.2)	3.3 (1.5)	2.2 (2.0)	3.2 (2.4)	16.4 (3.7)	−0.3 (1.1)	−0.0 (1.5)	−0.2 (1.8)	0.5 (2.6)
*p*-value		0.562	0.906	0.864	0.634	0.582	0.352	**<0.001**	**<0.001**	**<0.001**	**<0.001**

^1^ overall median of age was 65 years. T = Tinnitus; D = Depression; A = Anxiety; C = Cognition; BS = Baseline Score; CS = Change Score. Bold printing of *p*-values < 0.05.

**Table 2:** Results of linear regression models (coefficient; standard error of mean; two-sided *t*-test *p*-values, significance levels of 0.05 with *p*-values < 0.05 highlighted in bold; increase in coefficient of determination; upper and lower limits of the 95% confidence intervals).

	Coefficient	SE	*p*-Value	ɅR^2^	95% CI
***M*_1_ = cognition cfb**	**R^2^ = 0.25, F(7, 586) = 27.69, *p* < 0.0001**				
Constant	2.1483	0.5912	**0.0003**		[0.9871; 3.3094]
Treatment (*X*)	−3.1084	0.2593	**<0.0001**		[−3.6176; −2.5992]
Sex (*W*)	−0.4019	0.3376	0.2343		[−1.0649; 0.2611]
Interaction treatment with sex (*XW*)	0.5238	0.4862	0.2818	0.0015	[−0.4311; 1.4786]
Cognition baseline (*U_1_*)	−0.0804	0.0295	**0.0067**		[−0.1384; −0.0223]
Tinnitus baseline (*U_2_*)	−0.0376	0.0690	0.5861		[−0.1731; 0.0979]
Depression baseline (*U_3_*)	−0.1081	0.0564	**0.0558**		[−0.2188; 0.0027]
Anxiety baseline (*U_4_*)	0.0398	0.0477	0.4045		[−0.0539; 0.1336]
**M_2_ = depression cfb**	**R^2^ = 0.23, F(7, 586) = 24.42, *p* < 0.0001**				
Constant	0.9217	0.3197	**0.0041**		[0.2939; 1.5496]
Treatment (*X*)	−0.7611	0.1402	**<0.0001**		[−1.0365; −0.4858]
Sex (*W*)	−0.2145	0.1825	0.2404		[−0.5730; 0.1440]
Interaction treatment with sex (*XW*)	0.0734	0.2629	0.7802	0.0001	[−0.4429; 0.5897]
Cognition baseline (*U_1_*)	−0.0063	0.0160	0.6945		[−0.0376; 0.0251]
Tinnitus baseline (*U_2_*)	−0.0328	0.0373	0.3790		[−0.1061; 0.0404]
Depression baseline (*U_3_*)	−0.3461	0.0305	**<0.0001**		[−0.4060; −0.2862]
Anxiety baseline (*U_4_*)	0.0275	0.0258	0.2869		[−0.0232; 0.0782]
**M_3_ = anxiety cfb**	**R^2^ = 0.32, F(7, 586) = 39.25, *p* < 0.0001**				
Constant	1.3796	0.3413	**0.0001**		[0.7093; 2.0499]
Treatment (*X*)	−0.9245	0.1497	**<0.0001**		[−1.2184; −0.6305]
Sex (*W*)	−0.2450	0.1949	0.2091		[−0.6277; 0.1377]
Interaction treatment with sex (*XW*)	0.2369	0.2807	0.3990	0.0008	[−0.3143; 0.7881]
Cognition baseline (*U_1_*)	−0.0274	0.0171	0.1087		[−0.0609; 0.0061]
Tinnitus baseline (*U_2_*)	0.0274	0.0398	0.4923		[−0.0508; 0.1056]
Depression baseline (*U_3_*)	0.0609	0.0326	0.0618		[−0.0030; 0.1249]
Anxiety baseline (*U_4_*)	−0.4143	0.0276	**<0.0001**		[−0.4684; −0.3602]
**Y = tinnitus cfb**	**R^2^ = 0.36, F(13, 580) = 25.15, *p* < 0.0001**				
Constant	−0.2173	0.2654	0.4133		[−0.7387; 0.3040]
Treatment (*X*)	−0.6158	0.1328	**<0.0001**		[−0.8767; −0.3549]
Cognition cfb (*M_1_*)	0.0945	0.0226	**<0.0001**		[0.0502; 0.1389]
Depression cfb (*M_2_*)	0.1056	0.0405	**0.0093**		[0.0262; 0.1851]
Anxiety cfb (*M_3_*)	0.1350	0.0368	**0.0003**		[0.0627; 0.2073]
Sex (*W*)	0.1487	0.1489	0.3184		[−0.1438; 0.4412]
Interaction treatment with sex (*XW*)	0.1725	0.2409	0.4742	0.0006	[−0.3006; 0.6456]
Interaction treatment with cognition cfb (*XM_1_*)	−0.0087	0.0417	0.8354	<0.0001	[−0.0906; 0.0733]
Interaction treatment with depression cfb (*XM_2_*)	−0.0072	0.0753	0.9234	<0.0001	[−0.1552; 0.1407]
Interaction treatment with anxiety cfb (*XM_3_*)	0.0219	0.0647	0.7346	0.0001	[−0.1051; 0.1490]
Cognition baseline (*U_1_*)	0.0382	0.0131	**0.0036**		[0.0125; 0.0638]
Tinnitus baseline (*U_2_*)	−0.3033	0.0303	**<0.0001**		[−0.3627; −0.2439]
Depression baseline (*U_3_*)	0.0115	0.0281	0.6808		[−0.0436; 0.0666]
Anxiety baseline (*U_4_*)	0.0639	0.0252	**0.0115**		[0.0144; 0.1134]

*M_i_* = Mediator (i = 1, 2, 3); *U_j_* = Covariable (j = 1, 2, 3, 4); *X* = independent variable; *W* = moderator; *Y* = dependent variable; *XM_i_* = interaction of independent variable and mediator (*i* = 1, 2, 3); *XW* = interaction of independent variable and moderator; CI = Confidence interval; SE = standard error of mean; cfb = change from baseline; R^2^ = statistical measure that determines the proportion of variance in the dependent variable that can be explained by the regression model; F = F statistic; *p* = *p*-value; ɅR^2^ = increase of R^2^ compared to a regression model without moderator. Bold printing of *p*-values < 0.05.

**Table 3:** Conditional indirect treatment effects on tinnitus severity (effect size; bootstrap standard error of mean and upper and lower limits of 95% bootstrap confidence intervals with 95% bootstrap confidence intervals not overlapping with zero highlighted in bold; 5000 bootstrap samples for percentile bootstrap confidence intervals; index of moderation is the difference between conditional indirect effects.).

		Effect	Bootstrap SE	95% Bootstrap CI
1—Mediated by cognition cfb	Female	−0.2938	0.0896	**[−0.4779; −0.1218]**
	Male	−0.2219	0.1014	**[−0.4248; −0.0210]**
Index of moderated mediation		0.0719	0.1343	[−0.1928; 0.3312]
2—Mediated by depression cfb	Female	−0.0804	0.0374	**[−0.1624; −0.0161]**
	Male	−0.0677	0.0527	[−0.1741; 0.0321]
Index of moderated mediation		0.0127	0.0631	[−0.1098; 0.1396]
3—Mediated by anxiety cfb	Female	−0.1248	0.0423	**[−0.2164; −0.0502]**
	Male	−0.1079	0.0637	**[−0.2508; −0.0096]**
Index of moderated mediation		0.0169	0.0730	[−0.1368; 0.1501]

SE = standard error of mean; cfb = change from baseline; CI = confidence interval. Bold indicates statistical significance.

**Table 4:** Conditional direct treatment effects on tinnitus severity (effect size; standard error of mean; two-sided *t*-test *p*-value, significance levels of 0.05 with *p*-values < 0.05 highlighted in bold; upper and lower limits of 95% confidence intervals).

		Effect	SE	*p*-Value	95% CI
Conditional direct effects	Female	−0.6158	0.1328	**<0.0001**	[−0.8767; −0.3549]
	Male	−0.4433	0.2011	**0.0279**	[−0.8383; −0.0483]

SE = standard error of mean; CI = confidence interval. Bold printing of *p*-values < 0.05.

## Data Availability

Due to ethical reasons and in terms of data protection law, raw data cannot be shared. To the extent permitted by law, trial data required for the purposes of validation have already been disclosed in result reports on corresponding databases.
